# Some Improved Ratio, Product, and Regression Estimators of Finite Population Mean When Using Minimum and Maximum Values

**DOI:** 10.1155/2013/431868

**Published:** 2013-11-17

**Authors:** Manzoor Khan, Javid Shabbir

**Affiliations:** Department of Statistics, Quaid-i-Azam University, Islamabad 45320, Pakistan

## Abstract

Efficient estimation of finite population mean is carried out by using the auxiliary information meaningfully. In this paper we have suggested some modified ratio, product, and regression type estimators when using minimum and maximum values. Expressions for biases and mean squared errors of the suggested estimators have been derived up to the first order of approximation. The performances of the suggested estimators, relative to their usual counterparts, have been studied, and improved performance has been established. The improvement in efficiency by making use of maximum and minimum values has been verified numerically.

## 1. Introduction

Supplementary information in form of the auxiliary variable is rigorously used for the estimation of finite population mean for the study variable. Ratio and product estimators due to Cochran [1] and Murthy [2], respectively, are good examples when information on the auxiliary variable is incorporated for improved estimation of finite population mean of the study variable. When correlation between the study variable (*y*) and the auxiliary variable (*x*) is positive, ratio method of estimation is effective and when correlation is negative, product method of estimation is used. There are a lot of improvements and advancements in the construction of ratio, product, and regression estimators using the auxiliary information. For recent details, see Haq et al. [3], Haq and Shabbir [4], Yadav and Kadilar [5], Kadilar and Cingi [6], and Koyuncu and Kadilar [7] and the references cited therein.

The ratio method of estimation is at its best when the relationship between *y* and *x* is linear and the line of regression passes through the origin but as the line departs from origin, the efficiency of this method decreases. In practice, the condition that the line of regression passes through the origin is rarely satisfied and regression estimator is used for estimation of population mean. 

Let *U* = (*U*_1_, *U*_2_,…, *U*_*N*_) be a population of size *N*. Let (*y*_*i*_, *x*_*i*_) be the values of the study and the auxiliary variables, respectively, on the *i*th unit of a finite population.

Let us assume that a simple random sample of size *n* is drawn without replacement from *U* for estimating the population mean $\overset{-}{Y}=\sum_{i=1}^{N}y_{i}/N$. It is further assumed that the population mean $\overset{-}{X}=\sum_{i=1}^{N}x_{i}/N$ of the auxiliary variable *x* is known. The minimum say (*x*_min⁡_) and maximum say (*x*_max⁡_) values of the auxiliary variables are also assumed to be known. 

The variance of mean per unit estimator $\overset{-}{y}=\sum_{i=1}^{n}y_{i}/n$ is given by

(1) $$\begin{array}{c} V\left(\overset{-}{y}\right)=\lambda S_{y}^{2}, \end{array}$$

where *λ* = ((1/*n*)−(1/*N*)) and $S_{y}^{2}=(1/\left(N-1\right))\sum_{i=1}^{N}{\left(y_{i}-\overset{-}{Y}\right)}^{2}$.

Some time there exists unusually very large (say *y*_max⁡_) and very small (say *y*_min⁡_) units in the population. The mean per unit estimator is very sensitive to these unusual observations and as a result population mean will be either underestimated (in case the sample contains *y*_min⁡_) or overestimated (in case the sample contains *y*_max⁡_). To overcome the situation Sarndal [8] suggested the following unbiased estimator:

(2) $$\begin{array}{c} {\overset{-}{y}}_{s}=\{\begin{array}{cc} \overset{-}{y}+c\; & \text{if}\;\;\text{sample}\;\;\text{contains}\;\;y_{\min}\;\;\text{but}\;\;\text{not}\;\;y_{\max},\\ \overset{-}{y}-c\; & \text{if}\;\;\text{sample}\;\;\text{contains}\;\;y_{\max}\;\;\text{but}\;\;\text{not}\;\;y_{\min},\\ \overset{-}{y}\; & \text{for}\;\;\text{all}\;\;\text{other}\;\;\text{samples}, \end{array} \end{array}$$

where *c* is a constant.

The variance of ${\overset{-}{y}}_{s}$ is given by

(3) $$\begin{array}{c} V\left({\overset{-}{y}}_{s}\right)=\lambda S_{y}^{2}-\frac{2\lambda nc}{N-1}\left(y_{\max}-y_{\min}-nc\right). \end{array}$$

Further, $V({\overset{-}{y}}_{s})<V(\overset{-}{y})$ if 0 < *c* < (*y*_max⁡_ − *y*_min⁡_)/*n*. 

For, *c*_opt_ = (*y*_max⁡_ − *y*_min⁡_)/2*n*, variance of ${\overset{-}{y}}_{s}$ is given by

(4) $$\begin{array}{c} V{\left({\overset{-}{y}}_{s}\right)}_{\text{opt}}=V\left(\overset{-}{y}\right)-\frac{\lambda {\left(y_{\max}-y_{\min}\right)}^{2}}{2\left(N-1\right)}, \end{array}$$

which is always smaller than $V(\overset{-}{y})$.

The usual ratio and product estimators of population mean $\left(\overset{-}{Y}\right)$ are given by

(5) $$\begin{array}{c} {\overset{-}{y}}_{R}=\overset{-}{y}\left(\frac{\overset{-}{X}}{\overset{-}{x}}\right),\\ {\overset{-}{y}}_{P}=\overset{-}{y}\left(\frac{\overset{-}{x}}{\overset{-}{X}}\right), \end{array}$$

where $\overset{-}{y}=\sum_{i=1}^{n}y_{i}/n$ and $\overset{-}{x}=\sum_{i=1}^{n}x_{i}/n$ are the sample means of variables *y* and *x*, respectively.

The expressions for biases (*B*(·)), and mean square errors (*M*(·)), of the conventional ratio and product estimators, are given by

(6) $$\begin{array}{c} B\left({\overset{-}{y}}_{R}\right)≅\overset{-}{Y}\lambda \left(C_{x}^{2}-\rho_{yx}C_{y}C_{x}\right), \end{array}$$

(7) $$\begin{array}{c} B\left({\overset{-}{y}}_{P}\right)=\overset{-}{Y}\lambda \rho_{yx}C_{y}C_{x}, \end{array}$$

(8) $$\begin{array}{c} M\left({\overset{-}{y}}_{R}\right)≅{\overset{-}{Y}}^{2}\lambda \left(C_{y}^{2}+C_{x}^{2}-2\rho_{yx}C_{y}C_{x}\right), \end{array}$$

(9) $$\begin{array}{c} M\left({\overset{-}{y}}_{p}\right)≅{\overset{-}{Y}}^{2}\lambda \left(C_{y}^{2}+C_{x}^{2}+2\rho_{yx}C_{y}C_{x}\right), \end{array}$$

where $C_{y}=S_{y}/\overset{-}{Y}$ and $C_{x}=S_{x}/\overset{-}{X}$ are the coefficients of variation of *y* and *x*, respectively, *ρ*_*yx*_ = *S*_*yx*_/*S*_*y*_ *S*_*x*_ is the correlation coefficient between *y* and *x*, $S_{x}^{2}=\sum_{i=1}^{N}{\left(x_{i}-\overset{-}{X}\right)}^{2}/\left(N-1\right)$, and $S_{yx}=\sum_{i=1}^{N}\left(y_{i}-\overset{-}{Y}\right)\left(x_{i}-\overset{-}{X}\right)/\left(N-1\right)$ are the population variance and population covariance, respectively. 

Usual regression estimator is given by

(10) $$\begin{array}{c} {\overset{-}{y}}_{lr}=\overset{-}{y}+b\left(\overset{-}{X}-\overset{-}{x}\right), \end{array}$$

where *b* is the sample regression coefficient. 

The variance of the estimator ${\overset{-}{y}}_{lr}$ is given by

(11) $$\begin{array}{c} V\left({\overset{-}{y}}_{lr}\right)=\lambda S_{y}^{2}\left(1-\rho_{yx}^{2}\right). \end{array}$$

## 2. Proposed Estimators

Motivated by Sarndal [8], we extend this idea to estimators which make use of the auxiliary information for increased precision. It is well known that ratio and product estimators are used when *y* and *x* are positively and negatively correlated, respectively. We suggest estimator for each case separately as follows.

**Case 1 (positive correlation between *y* and *x*)**

When *y* and *x* are positively correlated, then with selection of a larger value of *x*, a larger value of *y* is expected to be selected and when smaller value of *x* is selected, selection of a smaller value of *y* is expected. So we define the following estimators:

(12) $$\begin{array}{c} {\hat{\overset{-}{Y}}}_{RC}=\frac{{\overset{-}{y}}_{c_{11}}}{{\overset{-}{x}}_{c_{21}}}\overset{-}{X}=\{\begin{array}{cc} \frac{\overset{-}{y}+c_{1}}{\overset{-}{x}+c_{2}}\overset{-}{X}, & \\ \frac{\overset{-}{y}-c_{1}}{\overset{-}{x}-c_{2}}\overset{-}{X}, & \\ \frac{\overset{-}{y}}{\overset{-}{x}}\overset{-}{X} & \end{array} \end{array}$$

and similarly

(13) $$\begin{array}{c} {\overset{-}{y}}_{lrC1}={\overset{-}{y}}_{c_{11}}+b\left(\overset{-}{X}-{\overset{-}{x}}_{c_{21}}\right), \end{array}$$

where $\left({\overset{-}{y}}_{c_{11}}=\overset{-}{y}+c_{1},{\overset{-}{x}}_{c_{21}}=\overset{-}{x}+c_{2}\right)$ if the sample contains *y*_min⁡_ and *x*_min⁡_; $\left({\overset{-}{y}}_{c_{11}}=\overset{-}{y}-c_{1},{\overset{-}{x}}_{c_{21}}=\overset{-}{x}-c_{2}\right)$ if the sample contains *y*_max⁡_ and *x*_max⁡_, and $\left({\overset{-}{y}}_{c_{11}}=\overset{-}{y},{\overset{-}{x}}_{c_{21}}=\overset{-}{x}\right)$ for all other combinations of samples.

**Case 2 (negative correlation between *y* and *x*)**

When *y* and *x* are negatively correlated then with selection of a larger value of *x*, a smaller value of *y* is expected to be selected and when smaller value of *x* is selected, a larger value of *y* is expected to be selected. Keeping these points in view, the following estimators are therefore suggested:

(14) $$\begin{array}{c} {\hat{\overset{-}{Y}}}_{PC}={\overset{-}{y}}_{c_{12}}\frac{{\overset{-}{x}}_{c_{22}}}{\overset{-}{X}}=\{\begin{array}{cc} \frac{\left(\overset{-}{y}+c_{1}\right)\left(\overset{-}{x}-c_{2}\right)}{\overset{-}{X}}, & \\ \frac{\left(\overset{-}{y}-c_{1}\right)\left(\overset{-}{x}+c_{2}\right)}{\overset{-}{X}}, & \\ \frac{\overset{-}{y}}{\overset{-}{x}}\overset{-}{X} & \end{array} \end{array}$$

and similarly

(15) $$\begin{array}{c} {\overset{-}{y}}_{lrC2}={\overset{-}{y}}_{c_{11}}+b\left(\overset{-}{X}-{\overset{-}{x}}_{c_{22}}\right), \end{array}$$

where $\left({\overset{-}{y}}_{c_{12}}=\overset{-}{y}+c_{1},{\overset{-}{x}}_{c_{22}}=\overset{-}{x}-c_{2}\right)$ if sample contains *y*_min⁡_ and *x*_max⁡_; ${\overset{-}{y}}_{c_{12}}=(\overset{-}{y}-c_{1},{\overset{-}{x}}_{c_{22}}=\overset{-}{x}+c_{2})$, if sample contains *y*_max⁡_ and *x*_min⁡_, and $\left({\overset{-}{y}}_{c_{12}}=\overset{-}{y},{\overset{-}{x}}_{c_{22}}=\overset{-}{x}\right)$ for all other combinations of samples.

To find the bias and mean square error of these suggested estimators, we first prove two theorems which will be used in subsequent derivations.

**Theorem 1**

If a sample of size *n* units is drawn from a population of size *N* units, then the covariance between ${\overset{-}{y}}_{c_{12}}$ and ${\overset{-}{x}}_{c_{12}}$, when they are positively correlated, is given by

(16)
Cov (y−c11,x−c21)=λSyx−nλN−1×[c1(xmax⁡−xmin⁡)+c2(ymax⁡−ymin⁡)  −2nc1c2].

**Proof**

Let us assume that *n* units have been drawn without replacement from a population of size *N*. Let *S*_*n*_ denote a sample space. We partition the whole sample space into three mutually exclusive and collectively exhaustive sets, that is, *S*_1_, *S*_2_, and *S*_3_ such that *S*_*n*_ = *S*_1_ ∪ *S*_2_ ∪ *S*_3_. Further *S*_1_ is the set of all possible samples which contains *y*_min⁡_ and *x*_min⁡_, and *S*_2_ consists of all samples which contains *y*_max⁡_ and *x*_max⁡_, and *S*_3_ = *S* − *S*_1_ − *S*_2_. The number of sample points in *S*_1_, *S*_2_, and *S*_3_ is given by $\left(\begin{array}{c} N-2\\ n-1 \end{array}\right)$, $\left(\begin{array}{c} N-2\\ n-1 \end{array}\right)$, and $\left(\begin{array}{c} N\\ n \end{array}\right)-2\left(\begin{array}{c} N-2\\ n-1 \end{array}\right)$, respectively.

By definition of covariance, we have

(17) Cov (y−c11,x−c21) =(Nn)−1[∑s∈S1(y−+c1−Y−)(x−+c2−X−) +∑s∈S2(y−−c1−Y−)(x−−c2−X−) +∑s∈S3(y−−Y−)(x−−X−)] =(Nn)−1[∑s∈S1(y−−Y−)(x−−X−) +∑s∈S2(y−−Y−)(x−−X−) +∑s∈S3(y−−Y−)(x−−X−) −c1{∑s∈S2(x−−X−)−∑s∈S1(x−−X−)} −c2{∑s∈S2(y−−Y−)−∑s∈S1(y−−Y−)} +c1c2{∑s∈S1+∑s∈S2}] =(Nn)−1[∑s∈S(y−−Y−)(x−−X−)]−(Nn)−1  ×[c1(xmax⁡−xmin⁡n)(N−2n−1)−c2(ymax⁡−ymin⁡n)(N−2n−1)+2c1c2(N−2n−1)] =Cov (y−,x−)−N−nN(N−1)  ×[c1(xmax⁡−xmin⁡)+c2(ymax⁡−ymin⁡) −2nc1c2] =λSyx−nλ(N−1)  ×[c1(xmax⁡−xmin⁡)+c2(ymax⁡−ymin⁡) −2nc1c2].

**Theorem 2**

If a sample of size *n* units is drawn from a population of size *N* units, then the covariance between ${\overset{-}{y}}_{c_{12}}$ and ${\overset{-}{x}}_{c_{22}}$, when they are negatively correlated, is given by

(18)
Cov
 (y−c12,x−c22)=λSyx+nλN−1 ×[c1(xmax⁡−xmin⁡)+c2(ymax⁡−ymin⁡)−2nc1c2].

The above Theorem 2 can be proved similarly as Theorem 1.

We define the following relative error terms.

Let $e_{0}=\left({\overset{-}{y}}_{c_{1}}-\overset{-}{Y}\right)/\overset{-}{Y}$ and $e_{1}=({\overset{-}{x}}_{c_{2}}-\overset{-}{X})/\overset{-}{X}$, such that

(19) $$\begin{array}{c} E\left(e_{0}\right)=E\left(e_{1}\right)=0, \end{array}$$

(20) $$\begin{array}{c} E\left(e_{0}^{2}\right)=\frac{\lambda }{{\overset{-}{Y}}^{2}}\left[S_{y}^{2}-\frac{2nc_{1}}{N-1}\left(y_{\max}-y_{\min}-nc_{1}\right)\right], \end{array}$$

(21) $$\begin{array}{c} E\left(e_{1}^{2}\right)=\frac{\lambda }{{\overset{-}{X}}^{2}}\left[S_{x}^{2}-\frac{2nc_{2}}{N-1}\left(x_{\max}-x_{\min}-nc_{2}\right)\right], \end{array}$$

(22) E(e0e1)=λY− X−[Syx−nN−1×{c2(ymax⁡−ymin⁡)+c1(xmax⁡−xmin⁡)−2nc1c2}].

Expressing ${\hat{\overset{-}{Y}}}_{RC}$ in terms of *e*'s, we have

(23) $$\begin{array}{c} {\hat{\overset{-}{Y}}}_{RC}=\overset{-}{Y}\left(1+e_{0}\right){\left(1+e_{1}\right)}^{-1}. \end{array}$$

Expanding and rearranging right-hand side of (23), to first degree of approximation, we have

(24) $$\begin{array}{c} \left({\hat{\overset{-}{Y}}}_{RC}-\overset{-}{Y}\right)≅\overset{-}{Y}\left(e_{0}+e_{1}-e_{0}e_{1}+e_{1}^{2}\right). \end{array}$$

Using (24), the bias of ${\hat{\overset{-}{Y}}}_{RC}$ is given by

(25) B(Y−^RC)≅λX−[R(Sx2−2nc2N−1(xmax⁡−xmin⁡−nc2))−{Syx−nN−1×(c2(ymax⁡−ymin⁡)+c1(xmax⁡−xmin⁡)−2nc1c2)}],

where $R=\overset{-}{Y}/\overset{-}{X}$.

Using (24), the mean square error of ${\hat{\overset{-}{Y}}}_{RC}$, to the first degree of approximation, is given by

(26) M(Y−^RC)≅λ[Sy2−2nc1N−1(ymax⁡−ymin⁡−nc1)+R2{Sx2−2nc2N−1(xmax⁡−xmin⁡−nc2)}−2R{Syx−nN−1×(c2(ymax⁡−ymin⁡)+c1(xmax⁡−xmin⁡)−2nc1c2)}]

or

(27) M(Y−^RC)≅M(y−R)−2λnN−1 ×[(c1−Rc2){(ymax⁡−ymin⁡)−R(xmax⁡−xmin⁡)−n(c1−Rc2)}].

To find optimum values of *c*_1_ and *c*_2_, we differentiate (27) with respect to *c*_1_ and *c*_2_ as

(28) ∂M(Y−^RC)∂c1=0⇒(ymax⁡−ymin⁡)−R(xmax⁡−xmin⁡) −2n(c1−Rc2)=0,∂M(Y−^RC)∂c2=0⇒(ymax⁡−ymin⁡)−R(xmax⁡−xmin⁡) −2n(c1−Rc2)=0

Here we have one equation with two unknowns so unique, solution is not possible, so we let *c*_2_ = (*x*_max⁡_ − *x*_min⁡_)/2*n*, and then *c*_1_ = (*y*_max⁡_ − *y*_min⁡_)/2*n*.

For optimum values of *c*_1_ and *c*_2_, the optimum mean square error of ${\hat{\overset{-}{Y}}}_{RC}$ is given by

(29) M(Y−^RC)opt≅M(y−R)−λ2(N−1) ×[(ymax⁡−ymin⁡)−R(xmax⁡−xmin⁡)]2.

Similarly the bias and mean square error or optimum mean square error of ${\hat{\overset{-}{Y}}}_{PC}$ are, respectively, given by

(30) B(Y−^PC)≅λX−[Syx−nN−1 ×{c2(ymax⁡−ymin⁡)+c1(xmax⁡−xmin⁡) −2nc1c2}],

(31) M(Y−^PC)≅M(y−P)−2λnN−1 ×[(c1+Rc2){(ymax⁡−ymin⁡)+R(xmax⁡−xmin⁡)−n(c1+Rc2)}].

For optimum values of *c*_1_ and *c*_2_, the optimum mean square error of ${\hat{\overset{-}{Y}}}_{PC}$ is given by

(32) M(Y−^PC)opt≅M(y−P)−λ2(N−1) ×[(ymax⁡−ymin⁡)−R(xmax⁡−xmin⁡)]2.

The variance of regression estimator ${\overset{-}{y}}_{lrC1}$ in case of positive correlation is given by

(33) V(y−lrC1)=V(y−lr)−2λnN−1 ×[(c1−βc2){(ymax⁡−ymin⁡)−β(xmax⁡−xmin⁡)−2n(c1−βc2)}],

where *β* = *ρ*_*yx*_(*S*_*y*_/*S*_*x*_) is the population regression coefficient of *y* on *x*.

For *c*_2_ = (*x*_max⁡_ − *x*_min⁡_)/2*n* and *c*_1_ = (*y*_max⁡_ − *y*_min⁡_)/2*n*, optimum variance of ${\overset{-}{y}}_{lrC1}$ is given by

(34) V(y−lrC1)opt=V(y−lr)−λ2(N−1) ×[(ymax⁡−ymin⁡)−β(xmax⁡−xmin⁡)]2.

For negative correlation, variance of the regression estimator ${\overset{-}{y}}_{lrC2}$ is given by

(35) V(y−lrC2)=V(y−lr)−2λnN−1 ×[(c1+βc2){(ymax⁡−ymin⁡)+β(xmax⁡−xmin⁡)−2n(c1+βc2)}].

For *c*_2_ = (*x*_max⁡_ − *x*_min⁡_)/2*n* and *c*_1_ = (*y*_max⁡_ − *y*_min⁡_)/2*n*, optimum variance of ${\overset{-}{y}}_{lrC2}$ is given by

(36) V(y−lrC2)opt=V(y−lr)−λ2(N−1) ×[(ymax⁡−ymin⁡)+β(xmax⁡−xmin⁡)]2.

So in general we can write $V{\left({\overset{-}{y}}_{lrC}\right)}_{\text{opt}}$ as

(37) V(y−lrC)opt=V(y−lr)−λ2(N−1) ×[(ymax⁡−ymin⁡)−|β|(xmax⁡−xmin⁡)]2.

## 3. Comparison

The conditions under which the suggested estimators ${\hat{\overset{-}{Y}}}_{RC}$, ${\hat{\overset{-}{Y}}}_{PC}$, and ${\overset{-}{y}}_{lrC}$ perform better than the usual mean per unit estimator and their usual counterpart is given below.

*(a) Comparison of Proposed Ratio Type Estimator.* A proposed estimator ${\hat{\overset{-}{Y}}}_{RC}$ will perform better than

 (i) mean per unit estimator (by (1) and (27)) if $V(\overset{-}{y})-M\left({\hat{\overset{-}{Y}}}_{RC}\right)>0$ or if

(38) ρyx>RSx2Sy−nRSySx(N−1)×[(c1−Rc2){(ymax⁡−ymin⁡)−R(xmax⁡−xmin⁡)−2n(c1−Rc2)}];

    (ii) usual ratio estimator (by (8) and (27)) if $M({\overset{-}{y}}_{R})-M\left({\hat{\overset{-}{Y}}}_{RC}\right)>0$ or if

(39) min⁡[Rc2,Rc2−{R(xmax⁡−xmin⁡)−(ymax⁡−ymin⁡)n}]<c1 <max⁡[Rc2,Rc2−{R(xmax⁡−xmin⁡)−(ymax⁡−ymin⁡)n}].

*(b) Comparison of Proposed Product Type Estimator.* A proposed product type estimator will perform better than

    (iii) mean per unit estimator if $V(\overset{-}{y})-M\left({\hat{\overset{-}{Y}}}_{PC}\right)>0$ (by (1) and (31)) or if

(40) ρyx<−RSx2Sy+nRSySx(N−1) ×[(c1+Rc2){(ymax⁡−ymin⁡)+R(xmax⁡−xmin⁡)−2n(c1+Rc2)}];

    (iv) usual product estimator if $M({\overset{-}{y}}_{P})-M\left({\hat{\overset{-}{Y}}}_{PC}\right)>0$ (by (9) and (31)) or if

(41) min⁡[−Rc2,−Rc2+(R(xmax⁡−xmin⁡)+(ymax⁡−ymin⁡)n)]<c1 <max⁡[−Rc2,−Rc2+(R(xmax⁡−xmin⁡)+(ymax⁡−ymin⁡)n)].

*(c) Comparison of Proposed Regression Type Estimator*. A proposed regression type estimator (positive correlation) will perform better than

    (v) mean per unit estimator if $V\left(\overset{-}{y}\right)-V\left({\overset{-}{y}}_{lrC1}\right)>0$ (by (1) and (33)) or if

(42) ρyx2>−2nSy2(N−1) ×[(c1−βc2){(ymax⁡−ymin⁡)−β(xmax⁡−xmin⁡)−2n(c1−βc2)}];

    (vi) usual regression estimator if $V\left({\overset{-}{y}}_{lr}\right)-V\left({\overset{-}{y}}_{lrC1}\right)>0$ (by (11) and (33)) or if

(43) min⁡[βc2,βc2−{β(xmax⁡−xmin⁡)−(ymax⁡−ymin⁡)n}]<c1 <max⁡[βc2,βc2−{β(xmax⁡−xmin⁡)−(ymax⁡−ymin⁡)n}].

A proposed regression type estimator (negative correlation) will perform better than

    (vii) mean per unit estimator if $V\left(\overset{-}{y}\right)-V\left({\overset{-}{y}}_{lrC2}\right)>0$ (by (1) and (35)) or if

(44) ρyx2>−2nSy2(N−1) ×[(c1+βc2){(ymax⁡−ymin⁡)+β(xmax⁡−xmin⁡)−2n(c1−βc2)}];

    (viii) usual regression estimator if $V\left({\overset{-}{y}}_{lr}\right)-V\left({\overset{-}{y}}_{lrC1}\right)>0$ (by (11) and (33)) or if

(45) min⁡[−βc2,−βc2+{β(xmax⁡−xmin⁡)−(ymax⁡−ymin⁡)n}]<c1 <max⁡[−βc2,−βc2+{β(xmax⁡−xmin⁡)−(ymax⁡−ymin⁡)n}].

*(d) Comparison of Suggested Estimators for Optimum Values of c*_1_ *and c*_2_ *with Usual Estimators.* For optimum values of *c*_1_ and *c*_2_, the proposed estimator will always perform better than usual mean per unit estimator and their usual counterparts (ratio, product and regression estimators).

## 4. Empirical Study

We consider the following datasets for numerical comparison.

**Population (Singh and Mangat [9, page 193])**

Let *y* be the milk yield in kg after new food and let *x* be the yield in kg before new yield. *N* = 27, *n* = 12, $\overset{-}{X}=10.41111$, $\overset{-}{Y}=11.25185$, *y*_max⁡_ = 14.8, *y*_min⁡_ = 7.9, *x*_max⁡_ = 14.5, *x*_min⁡_ = 6.5, *S*_*y*_^2^ = 4.103, *S*_*x*_^2^ = 4.931, *S*_*xy*_ = 4.454, and *ρ*_*yx*_ = 0.990.

**Population (Singh and Mangat [9, page 195])**

Let *y* be the weekly time (hours) spent in nonacademic activities and let *x* be the overall grade point average (4.0 bases). *N* = 36, *n* = 12, $\overset{-}{X}=2.798333$, $\overset{-}{Y}=14.77778$, *y*_max⁡_ = 33, *y*_min⁡_ = 6, *x*_max⁡_ = 3.82, *x*_min⁡_ = 1.81, *S*_*y*_^2^ = 38.178, *S*_*x*_^2^ = 0.3504, *S*_*xy*_ = −2.477, and *ρ*_*yx*_ = −0.6772.

**Population (Murthy [10, page 399])**

Let *y* be the area under wheat crop in 1964 and let *x* be the area under wheat crop in 1963. *N* = 34, *n* = 12, $\overset{-}{X}=208.882$, $\overset{-}{Y}=199.441$, *y*_max⁡_ = 634, *y*_min⁡_ = 6, *x*_max⁡_ = 564, *x*_min⁡_ = 5, *S*_*y*_^2^ = 22564.56, *S*_*x*_^2^ = 22652.05, *S*_*xy*_ = 22158.05, and *ρ*_*yx*_ = 0.980.

**Population (Cochran [11, page 152])**

Let *y* be population size in 1930 (in 1000) and *x* be the population size in 1920 (in 1000). *N* = 49, *n* = 12, $\overset{-}{X}=103.1429$, $\overset{-}{Y}=127.7959$, *y*_max⁡_ = 634, *y*_min⁡_ = 46, *x*_max⁡_ = 507, *x*_min⁡_ = 2, *S*_*y*_^2^ = 15158.83, *S*_*x*_^2^ = 10900.42, *S*_*xy*_ = 12619.78 and *ρ*_*yx*_ = 0.98.

The conditional values and results are given in Tables 1 and 2, respectively. 

For percentage relative efficiency (PRE), we use the following expression:

(46) PRE(y−i,y−)=V(y−)V(y−i)  or  M(y−i)×100 for i=R,P,RC,PC,lr,lrC.

## 5. Conclusion

From Table 2, it is observed that the ratio estimator ${\hat{\overset{-}{Y}}}_{RC}$ is performing better than ${\overset{-}{y}}_{R}$ in Populations 1, 3, and 4 because of positive correlation. The product estimator ${\hat{\overset{-}{Y}}}_{PC}$ is better than ${\overset{-}{y}}_{P}$ just in Population 2 because of negative correlation. The regression estimator ${\overset{-}{y}}_{lrC}$ outperforms than all other considered estimators and is preferable.

## Figures and Tables

**Table 1:** Numerical values of conditions (38)–(45).

Conditions	Population 1	Population 2	Population 3	Population 4
(38)	0.99 > 0.592	−0.667 > 0.2529*	0.980 > 0.478	0.981 > 0.525
(39)	0.214 < 0.287 < 0.360	0.442 < 1.125 < 1.807	22.23 < 26.16 < 30.094	22.92 < 24.5 < 26.07
(40)	0.99 < −0.592*	−0.667 < −0.253	0.980 < −0.4783*	0.981 < −0.525*
(41)	−0.360 < 0.287 < 0.93	−0.442 < 1.125 < 2.692	−22.238 < 26.16 < 74.57	−26.07 < 24.5 < 75.07
(42)	0.980 > 0.000	0.459 > 0.000	0.9605 > 0.000	0.963 > 0.000
(43)	0.273 < 0.287 < 0.301	−0.773 < 1.125 < −0.592*	22.78 < 26.16 < 29.549	24.36 < 24.5 < 24.63
(44)	0.980 > −1.913	0.459 > 0.272	0.9605 > −0.862	0.963 > −1.88
(45)	−0.30 < 0.287 < −0.15*	0.592 < 1.125 < 4.026	−30.63 < 26.16 < −22.78*	−24.36 < 24.5 < −21.2*

Note: *indicates that the condition is not satisfied.

**Table 2:** PRE of different estimators with respect to $\overset{-}{y}$.

Estimator	Population 1	Population 2	Population 3	Population 4
${\overset{-}{y}}_{R}$	1742.030	51.514	2501.29	2442.965
${\overset{-}{y}}_{P}$	21.053	175.210	26.383	23.998
${\overset{-}{y}}_{lr}$	5115.818	184.699	2536.131	2763.749
${\hat{\overset{-}{Y}}}_{RC}$	2319.293	54.325	2940.086	2502.692
${\hat{\overset{-}{Y}}}_{PC}$	21.117	212.639	26.424	24.004
${\overset{-}{y}}_{lrC}$	5249.673	208.254	2856.83	2764.336

## References

[1] Cochran WG (1940). The estimation of the yields of cereal experiments by sampling for the ratio to total produce. *The Journal of Agriculture Science*.

[2] Murthy MN (1964). Product method of estimation. *Sankhya A*.

[3] Haq A, Shabbir J, Gupta S (2013). Improved exponential ratio type estimators in stratified sampling. *Pakistan Journal of Statistics*.

[4] Haq A, Shabbir J (2013). Improved family of ratio estimators in simple and stratified random sampling. *Communication in Statistics: Theory and Methods*.

[5] Yadav SK, Kadilar C (2013). Improved class of ratio and product estimators. *Applied Mathematics and Computation*.

[6] Kadilar C, Cingi H (2006). Improvement in estimating the population mean in simple random sampling. *Applied Mathematics Letters*.

[7] Koyuncu N, Kadilar C (2009). Ratio and product estimators in stratified random sampling. *Journal of Statistical Planning and Inference*.

[8] Sarndal CE (1972). Sample survey theory vs general statistical theory: estimation of the population mean. *International Statistical Institute*.

[9] Singh R, Mangat NS (1996). *Elements of Survey Sampling*.

[10] Murthy MN (1967). *Sampling Theory and Methods*.

[11] Cochran WG (1977). *Sampling Techniques*.

